# Influence of high altitude after a prior ascent on physical exhaustion during cardiopulmonary resuscitation: a randomised crossover alpine field experiment

**DOI:** 10.1186/s13049-023-01132-7

**Published:** 2023-10-24

**Authors:** Maximilian Niederer, Katharina Tscherny, Josef Burger, Bettina Wandl, Verena Fuhrmann, Calvin L. Kienbacher, Wolfgang Schreiber, Harald Herkner, Dominik Roth, Alexander Egger

**Affiliations:** 1 Department of Anaesthesiology and Intensive Care Medicine, Hospital Scheibbs, Eisenwurzenstraße 26, 3270 Scheibbs, Austria; 2 Mountain Rescue Service Austria, Baumgasse 129, 1030 Vienna, Austria; 3 Department of Emergency Medicine, Medical University of Vienna, Spitalgasse 23, 1090 Vienna, Austria; 4 Department of Paediatrics, Hospital Lienz, Emanuel von Hibler-Straße 5 A, 9900 Lienz, Austria; 5 Institute of Nursing Science, Department of Nursing Science and Gerontology, UMIT TIROL – Private University for Health Sciences and Health Technology, Hall in Tyrol, Austria

**Keywords:** Cardiopulmonary resuscitation, High altitude, Physical exhaustion, Mountain medicine, Basic life support

## Abstract

**Background:**

Performing cardiopulmonary resuscitation (CPR) inevitably causes significant physical, as well as psychological stress for rescuers. Physical activity at high altitude, a hypobaric and hypoxic environment, similarly adds to the level of stress and causes multiple physiological changes. Continuous measurement of pulse rate serves as an objective measure of fatigue during CPR. We therefore aimed to investigate rescuers’ heart rates as a measure of physical strain during CPR in a high-altitude alpine environment to provide a better understanding of the physiological changes under these very special conditions.

**Methods:**

Twenty experienced mountaineers performed basic life support (BLS) on a manikin for 16 min, both at baseline altitude and at high altitude (3454 m) following a quick and exhausting ascent over 1200 m. Sequence of scenarios was randomised for analysis. Heart rate was continuously measured and compared between baseline and high altitude by absolute differences and robust confidence intervals.

**Results:**

During CPR at baseline, the average heart rate increased from 87 bpm (SD 16 bpm) to 104 bpm [increase 17 bpm (95% CI 8.24–24.76)], compared to an increase from 119 bpm (SD 12 bpm) to 124 bpm [increase 5 bpm (95% CI − 1.59 to 12.19)] at high altitude [difference between two groups 32 bpm (95% CI 25–39)]. Differences between periods of chest compressions and ventilations were very similar at baseline [19 bpm (95%CI 16.98–20.27)] and at high altitude [20 bpm 95% CI 18.56–21.44)], despite starting from a much higher level at high altitude. The average heart rates of rescuers at high altitude at any point were higher than those at baseline at any other point.

**Conclusion:**

Performing BLS CPR causes exhaustion both at base level and at a high altitude. A further increase during CPR might imply a physiological reserve for adapting to additional physical exertion at high altitude. Phases of ventilation are much needed recovery-periods, but heart rates remain very high. Subjective measures of exhaustion, such as the BORG-scale, might lead to rescuers’ overestimation of their own performance.

## Background

Performing cardiopulmonary resuscitation (CPR) inevitably causes significant physical [1] as well as psychological [2] stress for rescuers. This stress is inversely related to the ability to perform high quality CPR.

The physiological effects of CPR on rescuers’ physiology under “standard conditions” (at sea level) are quite well understood: after performing chest compressions, 79% of rescuers reported that they were suffering from subjective fatigue throughout the duration of the CPR. McDonald et al. [1] were able to objectively measure fatigue and found an increase during CPR. These data formed the basis for the recommendation to take turns in performing chest compressions every two minutes as part of the ERC Guidelines [3]. Hong et al. investigated the relationship between fatigue and heart rate. They found a strong correlation between the two, with a steady increase in heart rate during CPR. At the beginning of a new ventilation cycle the heart rate of the person performing chest compressions spiked, followed by a decrease during ventilation until the beginning of the next cycle of chest compressions [4].

Physical activity at high altitude, a hypobaric and hypoxic environment, similarly adds to the level of stress and causes multiple physiological changes [5–7]. The increase in popularity of alpine sports in recent times has led to a notable rise in cardiac incidents at high altitude, and this trend is likely to continue [8].

The physical strain encountered during alpine rescue operations, especially those involving resuscitation efforts, comprises two parts: the strain of mountaineering itself, i.e., a rapid ascent to the victim, and the strain of performing CPR in a hypobaric and hypoxic environment.

Although there is extensive literature on physical stress caused by performing CPR at sea level, little is known about the physical conditions during alpine rescue operations. Our study was dedicated to addressing this gap in knowledge. Continuous measurement of pulse rate has already been used by others [9, 10] as an objective measure of fatigue during CPR, and might be superior [9] to measurement of subjectively felt fatigue [1, 11]. However, no such measurement of fatigue has yet been performed during a realistic scenario at high altitude.

We therefore aimed to investigate rescuers’ heart rates as a measure of physical strain during such rescue operations in a high-altitude alpine environment. We hypothesized that there would be a significant change in heart rate during CPR between baseline and high altitude.

## Methods

### Study design

This was a prospective, experimental randomised crossover field experiment (A-B-A sequence design) in an alpine environment. Details on the study design have been previously published [12]. In the previous publication, we analysed differences in the quality of CPR between baseline (673 m) and high altitude (3450 m). We now focus on differences in physical strain encountered by the rescuers.

The study population comprised 20 members of the Austrian Mountain Rescue Service and the Austrian Army alpine branch, trained in basic life support (BLS) according to ERC guidelines [13]. Those participants formed ten teams of two rescuers each.

The subjects were experienced mountaineers, but no professional climbers. They featured skills and levels of physical training typical of members of the Austrian Mountain Rescue Service, an organization of 12,000 volunteers operating 300 rescue stations, dedicated to rescuing victims of mountain emergencies. Participants therefore represented potential rescuers most likely to be in a high-altitude emergency.

The study setting consisted of a base level at 673 m above sea level, and a quick ascent from a base camp over 1213 m in altitude to a mountain shelter at 3454 m.

After completing informed consent, the study subjects participated in a fitness-questionnaire (FFB-MOT) [14], a modified nine-hole peg test (NHPT) for dexterity [15], as well as measurement of their vital signs (blood pressure, heart rate and oxygen saturation.

Participants then performed 30:2 BLS CPR including bag-valve-mask ventilation (BVM) for 16 min on a training manikin (Laerdal Resusci Anne First Aid QCPR, Stavanger Norway) with a feedback device (M-Series, ZOLL-Chelmsford, MA), switching roles every two minutes. Hence each of the participants had to perform a total of eight rounds of chest compressions and eight rounds of BVM, respectively. Throughout the whole scenario we measured the participants’ heart rates using a breast heart rate sensor and pulse watch (Polar Vantage V, heart rate sensor H7) at an interval of every second as well as the quality of the CPR itself (SimPad PLUS with SkillReporter, Laerdal, Stavanger Norway). In addition, we evaluated subjectively felt fatigue every two minutes during CPR using the Borg CR-10 Scale [16], combining both objective and subjective measures of fatigue. At the end of the scenario, we repeated the measurement of vital signs and the modified NHPT.

On the second day, participants were brought to a base camp at 2241 m and performed a quick ascent to 3454 m. Immediately before departure, vital signs were measured, and backpacks were weighed.

During the whole time of the ascent, the participants wore the pulse watch with the heart rate sensor. After arriving at the “Adlersruhe” at an altitude of 3454 m, all measurements were repeated, and participants immediately performed BLS CPR for 16 min.

**Fig. 1:**
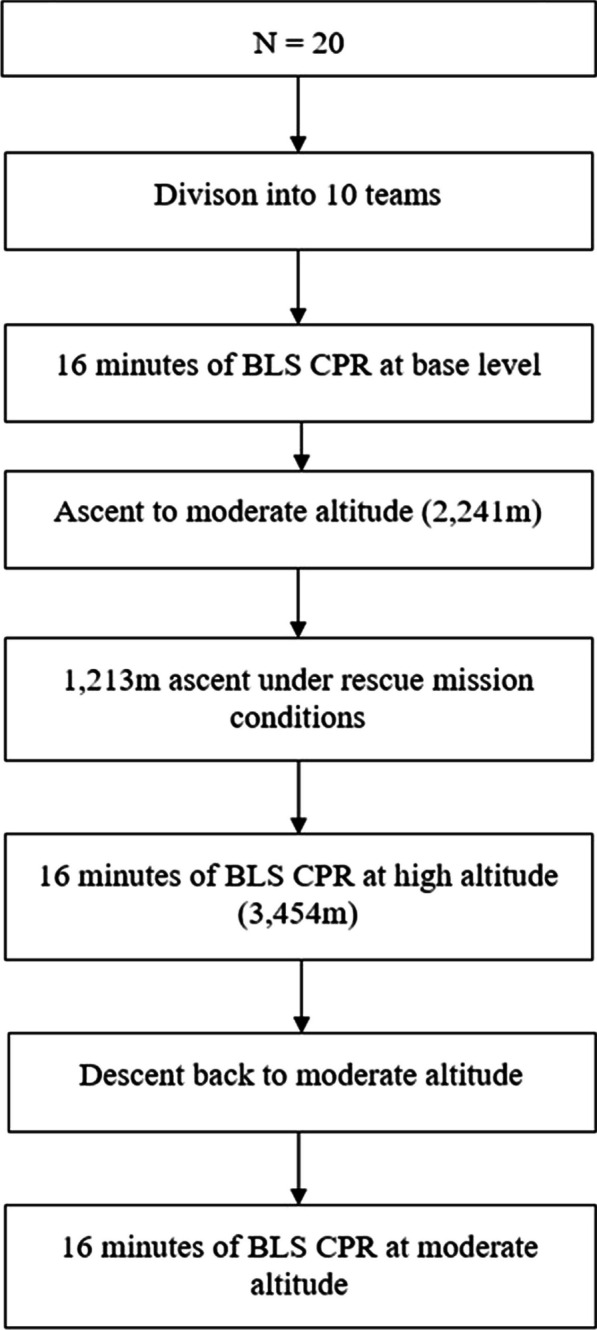
Flowchart of the study design

Finally, participants returned to baseline height at a moderate speed. They rested for one hour, after which all procedures (measurements and CPR) were performed for a third and final time, exactly as before. This was done to control for possible learning effects on CPR quality (Fig. 1).

### Analysis

Sample size calculations were based on the primary study outcome, CPR quality [12], resulting in 20 participants. For this secondary analysis, we calculated that given those 20 participants, we would be able to show a minimal difference of 4 bpm (SD 6 bpm) at a power of 90%, with a probability of error of the first kind of 5%. (The given sample size of 20 results in a required effect size dz of at least 0.67 for a paired t-test to reject the null hypothesis of no difference between baseline and high altitude with a one-tailed alpha of 0.05 at a power of 0.9. The aforementioned difference in heart rate of at least 4 bmp (SD 6 bpm) represents the smallest possible difference between groups with an effect size of at least 0.67. We used G*Power 3.1.9.7 for those calculations.

We tabulated all results by “high altitude” and “baseline altitude” measurements. To control for possible learning effects, we randomised participants to use the baseline altitude data from either before (“baseline altitude–high altitude”) or after (“high altitude–baseline altitude”) the high-altitude portion of the study, discarding the other baseline measurements.

We display results as absolute and relative frequencies, or as mean and standard deviation or median and interquartile range, as appropriate. For comparisons, we calculated absolute differences with robust confidence intervals. In addition, the paired sample t-test was performed. A *p*-value of < 0.05 was generally considered statistically significant. We used STATA 17 (StataCorp, College Station, TX) for all analyses.

## Results

### Study population

As described above, 20 volunteers participated in the field study. Table 1 shows the characteristics of the study subjects.

**Table 1:** Study population

	Total	Male	Female
Number of subjects	20	17 (85%)	3 (15%)
Age (years)	38 (SD 12)	40 (SD 12)	27 (SD 0)
Height (cm)	178 (SD 6)	180 (SD 5)	167 (SD 6)
Weight (kg)	74 (SD 8)	76 (SD 7)	63 (SD 6)
BMI (kg/m^2^)	23 (SD 2)	23 (SD 2)	23 (SD 1)
Smoker	2 (10%)	2 (12%)	0 (0%)
Weight of backpack (kg)	10 (SD 3)	11 (SD 2)	7 (SD 1)

SD, standard deviation

Table 2 shows the vital signs of the participants before and after CPR. Heart rates before CPR at high altitude were noticeably higher than in previous studies, where participants did not perform any physical effort, such as ascent, before CPR [17].

**Table 2:** Vital signs at baseline and high altitude, all values are mean (standard deviation)

	Baseline	High altitude
Heart rate (bpm)		
Before CPR	87 (16)	119 (12)
After CPR	84 (16)	121 (18)
Systolic blood pressure (mmHg)		
Before CPR	134 (10)	136 (14)
After CPR	139 (13)	129 (9)
Diastolic blood pressure (mmHg)		
Before CPR	78 (10)	83 (9)
After CPR	81 (11)	81 (8)
SpO2 (%)		
Before CPR	97 (2)	87 (3)
After CPR	98 (1)	88 (4)
BORG-Scale		
Before CPR	1.8 (1.0)	2.4 (0.8)
End of CPR	2.7 (1.1)	2.6 (0.8)

bpm, beats per minute

### Ascent to high altitude

The weather condition during our study were quite well without any kind of rain, fog or strong winds. The average duration of the ascent to high altitude was 2 h 58 min. (SD 40 min). The average heart rate during the ascent was 146 bpm (SD 11 bpm), ranging from 126 to 176 bpm. The highest observed peak heart rate was 220 bpm. In comparison, the average resting heart rate just before the ascent was 79 bpm (SD 12 bpm). On arrival at high altitude, we measured an average heart rate of 126 bpm (SD 47). This corresponds to an average increase of 47 bpm (95% CI 40–52).

### CPR at high altitude

**Fig. 2:**
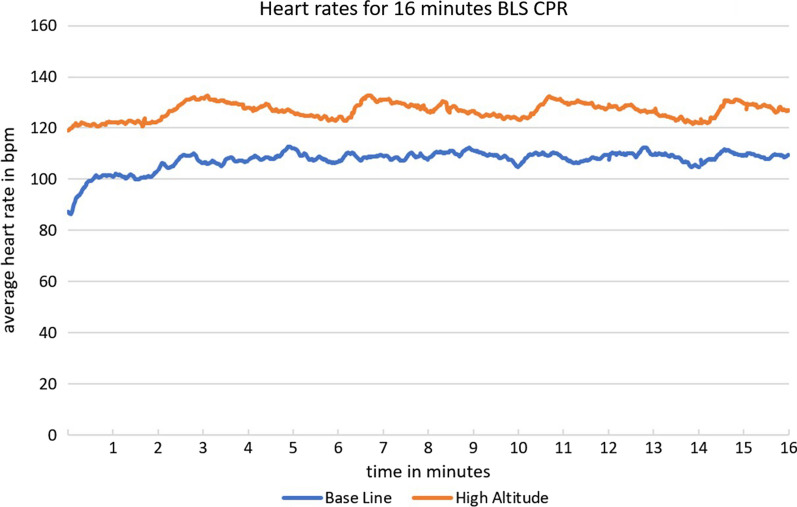
Heart rates for 16 min of BLS CPR

Figure 2 shows the average heart rates during the 16 min of CPR at base level and at high altitude. The average heart rate of rescuers at high altitude at any point was higher than that at baseline at any other point. This means that even during ventilation, rescuers had a higher heart rate at high altitude, than they had during chest compressions at baseline.

**Fig. 3:**
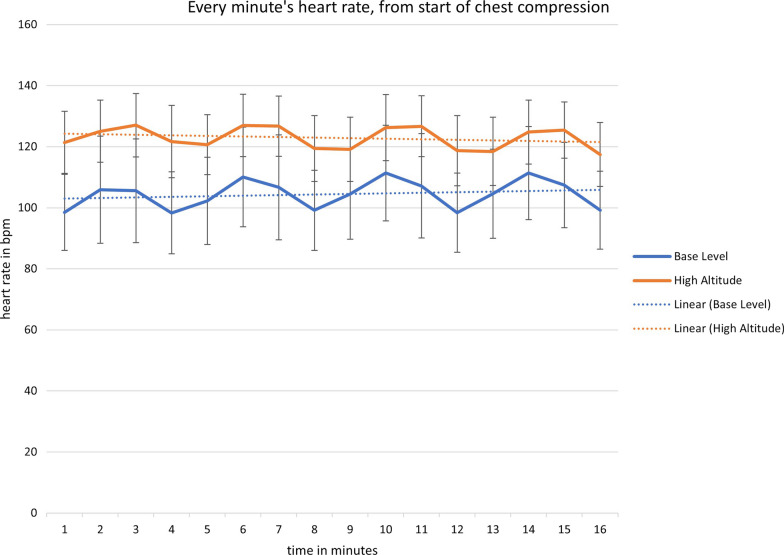
Minute by minute heart rate, from the start of chest compression

**Fig. 4:**
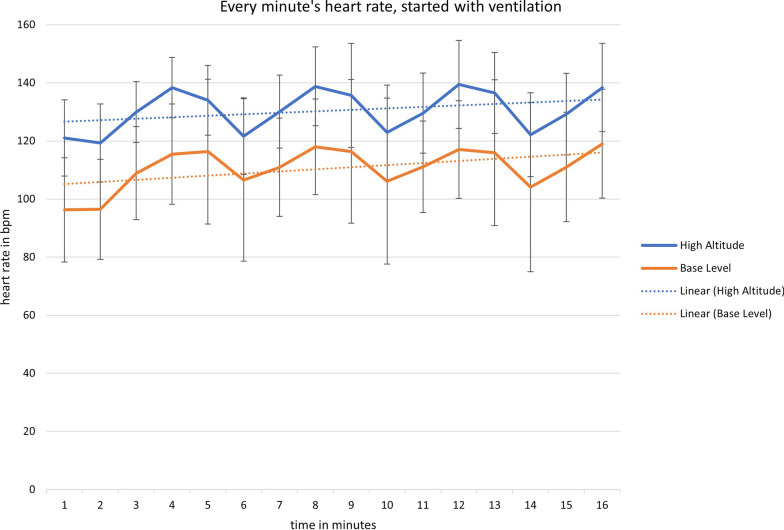
Minute by minute heart rate, starting with ventilation

Because CPR was performed in teams of two, half of the participants started CPR with chest compressions, whereas the other half started with ventilations. Figures 3 and 4 show heart rates for those starting with chest compressions, and those starting with ventilations, respectively. Whereas those starting with ventilations initially had lower heart rates, as expected, the general patterns of heart rates showed no differences between those two groups: both at baseline level and at high altitude, heart rate increased during chest compressions and then decreased again during ventilation.

**Fig. 5:**
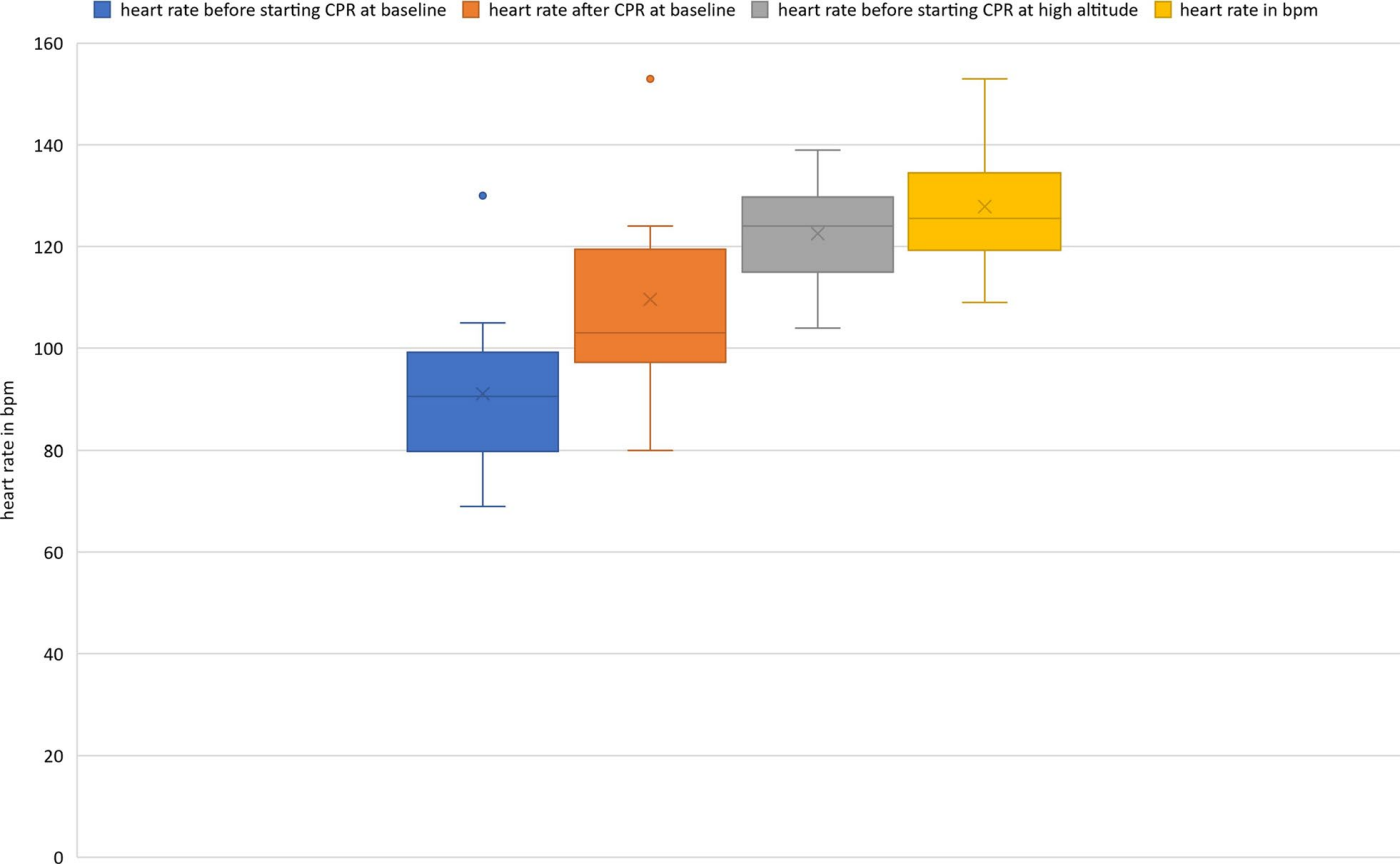
Pre-post comparison of the rescuers’ heart rate

After 16 min of CPR, heart rate had significantly increased, however the increase at baseline level was much more pronounced [from 87 bpm (SD 16 bpm) to 104 bpm, increase 17 bpm (95% CI 8.24–24.76)] than at high altitude [from 119 bpm (SD 12 bpm) to 124 bpm, increase 5 bpm (95%CI − 1.59 to 12.19)]. Interestingly, the average heart rate after 16 min of CPR at baseline level was still lower than that before even starting CPR at high altitude (see Fig. 5).

Surprisingly subjective measures of exhaustion (BORG-scale) at the end of CPR showed no difference between baseline and high-altitude (2.7 (SD 1.1) vs. 2.6 (SD 0.8), *p* > 0.05).

## Discussion

In our study, we analysed the impact of a quick ascent (1213 m of height) to high altitude (3454 m) on rescuers’ heart rates during BLS CPR. We found that even for well-trained mountaineers, CPR at high-altitude is extremely exhausting, and heart rates even at the beginning of CPR or during ventilation phases are significantly higher at high altitude, compared to baseline. Heart rates during CPR increased profoundly, both at baseline and from an already very high starting point at high altitude. This might imply that our volunteers had a physiological reserve to adapt to further physical exertion at high altitude. Subjective measures of exhaustion, such as the BORG-scale, might however lead to rescuers’ overestimation of their own performance. Exhaustion at occurs much quicker at high altitude, and guidelines should take account of this.

### Heart rates at the beginning of CPR

At baseline level, just before starting CPR, we started with an average heart rate of 87 bpm, which is very similar to the 88 bpm in the study of Hong et al. [4], who used a similar study population (20 male medical doctors, average 28 years of age).

On the other hand, heart rate before starting at high altitude was very high at 119 bpm in our study. In comparison, Narahara et al. [17], observed an average heart rate of only 86 bpm at high altitude (3700 m). However, participants in this study were transported to high altitude by car, without any physical effort before performing CPR. This might not be feasible in many mountainous settings, where the emergency location cannot be reached by car. Another possibility is the use of helicopters, as studied by Wang et al. [18]. Participants had an average heart rate of 94 bpm at 3100 m, before starting CPR. However, in addition to being brought to the location by helicopter, they had a resting period of 6 h before starting CPR, which would not be possible in a real-world scenario. Even the use of helicopters is often very limited, due to weather conditions and the situation at the emergency site.

In our study, just as in a genuine emergency situation, CPR was performed following exhausting activity and without any possibility for acclimatisation. This is mirrored by an impressive decrease in peripheral oxygen saturation (SpO2) from 97% (SD 2%) at baseline to 87 (SD 3%) at high altitude (*p* < 0.01). In addition to reduced oxygen partial pressure, the sympathetic drive in the first few days at high altitude leads to increased heart rate. This effect is directly proportional to altitude, and usually diminishes after a few days [6].

### Heart rates during CPR

During CPR at baseline, we observed a steep increase from a relaxed resting heart rate to about 100 bpm. This effect is well known, and represents the adaption to physical exhaustion of performing CPR [4]. At high altitude, even if participants already started at an average of about 120 bpm, the profile of the curves still mirrored the trend at baseline, with a further increase in heart rate during chest compressions.

Usually, periods of ventilation are used as a resting period during two-person CPR [4], and current guidelines recommend switching roles every two minutes, based on sea-level data. We found, however, that heart rate during ventilation at high altitude was still higher than that during chest compressions at baseline altitude.

Based on our findings, the current CPR guidelines might need to be adapted for high altitude environments. There already exist adaptions to the ‘universal CPR algorithm’, however those only apply to special circumstances of cardiac arrest (such as hypothermia or avalanches) [3]. Given the significant physiological changes rescuers undergo at high altitude, future studies should investigate whether more frequent (e.g.: every minute) changes between rescuers performing chest compressions should be warranted at high altitude. Given the limited use of subjective measures of exhaustion at high altitude, guidelines should also stress the role of feedback devices, possible use of pulse watches (which are already worn by many mountaineers) to monitor for exhaustion, as well as mechanical CPR devices in those settings.

### Limitations

This study was conducted as a manikin study, with the concomitant limitations of that study design, and might not fully replicate the complexities of real-life rescue operations, including emotional stressors. The subjects of interest in our study were, however, the rescuers, not the patients, reducing the downsides of the manikin-design. In addition, the study was performed in a real world setting. Nevertheless, different settings (even higher altitude, different weather or terrain) might lead to different findings.

Furthermore, our study population consisted of very well trained mountaineers and might not be representative of other groups. Again, we believe that our population quite well represented those who would have to perform CPR at high altitude.

## Conclusions

It is well known that performing CPR requires strong physical effort. Our results indicate that this is even more true during a realistic rescue scenario at high altitude. Heart rates are already quite high at the start of CPR at high altitude, due to the exhausting ascent and reduced oxygen partial pressure. Nevertheless, they increase even further during CPR, implying a physiological reserve to adapt to further physical exertion at high altitude. Phases of ventilation are much needed recovery-periods, but heart rates remain very high. Subjective measures of exhaustion, such as the BORG-scale, do not appear to accurately represent exhaustion at high altitude, possibly leading to rescuers’ overestimation of their own performance.

## Data Availability

The datasets used and/or analysed during the current study are available from the corresponding author on reasonable request.

## Abbreviations

95% CI: 95% Confidence interval
BLS: Basic life support
BMI: Body mass index
bpm: Beats per minute
BVM: Bag valve mask
CPR: Cardiopulmonary resuscitation
ERC: European Resuscitation Council
FFB-MOT: Fitness questionnaire
NHPT: Modified nine hole peg test
SD: Standard deviation
